# Size Is the Major Determinant of Pumping Rates in Marine Sponges

**DOI:** 10.3389/fphys.2019.01474

**Published:** 2019-12-11

**Authors:** Teresa Maria Morganti, Marta Ribes, Gitai Yahel, Rafel Coma

**Affiliations:** ^1^Max Planck Institute for Marine Microbiology, HGF MPG Joint Research Group for Deep-Sea Ecology and Technology, Bremen, Germany; ^2^Department of Marine Biology and Oceanography, Institut de Ciències del Mar (ICM-CSIC), Barcelona, Spain; ^3^The Faculty of Marine Science, Ruppin Academic Center, Michmoret, Israel; ^4^Department of Marine Ecology, Centre d’Estudis Avançats de Blanes (CEAB-CSIC), Girona, Spain

**Keywords:** Porifera, pumping rate, environmental factors, size, seasonality, allometric scaling, high-low microbial-abundance sponges

## Abstract

Sponges play an important ecological function in many benthic habitats. They filter large volumes of water, retain suspended particles with high efficiency, and process dissolved compounds. Nevertheless, the factors that regulate sponge pumping rate and its relation to environmental factors have been rarely studied. We examined, *in situ*, the variation of pumping rates for five Mediterranean sponge species and its relationship to temperature, particulate food abundance and sponge size over two annual cycles. Surprisingly, temperature and food concentration had only a small effect on pumping rates, and the seasonal variation of pumping rates was small (1.9–2.5 folds). Sponge size was the main determinant of the specific pumping rate (pumping normalized to sponge volume or mass). Within the natural size distribution of each species, the volume-specific pumping rate [PR*_V_*, ml min^−1^ (cm sponge)^−3^] decreased (up to 33 folds) with the increase in sponge volume (*V*, cm^3^), conforming to an allometric power function (PR*_V_* = *aV^b^*) with negative exponents. The strong dependence of the size-specific pumping rate on the sponge size suggests that the simplistic use of this value to categorize sponge species and predict their activity may be misleading. For example, for small specimens, size-specific pumping rates of the two low-microbial-abundance (LMA) species (allometric exponent *b* of −0.2 and −0.3) were similar to those of two of the high-microbial-abundance (HMA) species (*b* of −0.5 and −0.7). However, for larger specimens, size-specific pumping rates were markedly different. Our results suggest that the pumping rate of the sponges we studied can be approximated using the measured allometric constants alone in conjunction with surveys of sponge abundance and size distribution. This information is essential for the quantification of *in situ* feeding and respiration rates and for estimates of the magnitude of sponge-mediated energy and nutrient fluxes at the community level. Further work is required to establish if and to what extent the low seasonal effect and the strong size dependency of pumping rate can be generalized to other sponges and habitats.

## Introduction

Sponges filter a large volume of water daily, processing up to 35 ml min^−1^ (cm sponge)^−3^ (Weisz et al., 2008) and removing ultra-planktonic cells (<10 μm) with high retention efficiency (Pile et al., 1996; Coma et al., 2001; Yahel et al., 2007). Where sponges abound, they contribute considerably to nutrient import and cycling acting as a sink for organic matter and source of dissolved nutrients to the adjacent benthic community (Maldonado et al., 2012, 2016; Pawlik and McMurray, 2019). Sponge role in organic matter cycling has been equated to that of the microbial loop, by removing dissolved organic matter from the water column and making it available in the form of detritus to higher trophic levels (de Goeij et al., 2013; Rix et al., 2016a,b, 2017).

Marine sponges are distributed worldwide, from polar to the tropic regions, from the shallows to the deep sea (Van Soest et al., 2018). Under projected climate change scenarios, sponges will likely increase in abundance (Bell et al., 2013, 2018), leading to a cascading effect of sponge filtration on ecosystem processes (Pawlik et al., 2016). Proper assessment of the volume of water pumped by sponges in nature is crucial for the determination of the magnitude of sponge-mediated energy and nutrient fluxes between the pelagic and the benthic realm.

The sponge body is specialized for suspension feeding through a unique and highly vascularized canal system. The water enters the sponge body through small apertures called ostia (normally 20–60 micron; Simpson, 1984) that cover most of the sponge outer surface. Pumped water is drawn into the inhalant canals due to the slightly negative pressure created by the movement of choanocyte flagella gathered in the choanocyte chambers (Asadzadeh et al., 2019). The latter constitute the basic pumping units operating in parallel (Larsen and Riisgård, 1994), and the volume of water pumped is directly correlated to their density (Massaro et al., 2012). The capture of small particles occurs in the choanocyte chambers, while larger particles are trapped in the branching inhalant canals. After passing the chambers, the water leaves through the exhalant canals that merge into the excurrent apertures (oscula).

Active pumping may be complemented by a passive or induced flow generated by the ambient current flowing over the sponge and inducing a gradient of pressure across the sponge wall (Vogel, 1977). However, induced flow only occurs under very high ambient current and only for sponges with very thin walls and short canal systems that minimize the internal drag (Leys et al., 2011; Ludeman et al., 2017). The actual cost of pumping is still unclear: laboratory-based studies suggest that pumping is a low-cost process, accounting for <1% of the sponge metabolic expenditure (e.g., Riisgård et al., 1993; Riisgård and Larsen, 1995, 2010), while more recent works suggest that pumping may be an expensive process (e.g., Hadas et al., 2008; Leys et al., 2011; Ludeman et al., 2017). As sponge respiration, feeding, excretion, and reproduction are all mediated by the water they filter, factors that regulate sponge pumping rates are crucial for the understanding of basic sponge physiology. Information on the natural variation of sponge pumping is further important for the calculation of *in situ* feeding and excretion to estimate the magnitude of sponge-mediated energy and nutrient fluxes (e.g., Kahn et al., 2015).

Sponge pumping activity has been observed to be highly variable among species and individuals. While some species may maintain rather constant pumping activity, periodical interruptions of water transport may occur randomly through the population and irregularly among individuals (Reiswig, 1971; Bell et al., 1999; McMurray et al., 2014; Strehlow et al., 2016). Sponge pumping rate may vary from 0.3 ml min^−1^ (cm sponge)^−3^ in *Aplysina lacunosa* (Gerrodette and Flechsig, 1979) to 35 ml min^−1^ (cm sponge)^−3^ in *Callyspongia plicifera* (Weisz et al., 2008). Weisz et al. (2008) suggested that the dichotomy between high (HMA) and low (LMA) microbial-abundance species is also reflected in their specific pumping rate, with LMA sponges generally showing higher specific pumping rates than HMA sponges.

Knowledge of the factors that regulate pumping rates under natural conditions is scarce, and the most comprehensive study of *in situ* sponge pumping dynamics was performed on large Caribbean sponges by Henry Reiswig almost half century ago (Reiswig, 1971). Reiswig observed changes in pumping activity in Demospongiae and recorded pumping behaviors over long periods. Variations in pumping activity were related to temperature changes, storm season, and behavioral patterns (i.e., intrinsically generated or day-night cycles). Unlike the *in situ* and direct methods employed by Reiswig, many of the studies that followed applied indirect techniques on confined animals under laboratory conditions (Gerrodette and Flechsig, 1979; Frost, 1980; Riisgård et al., 1993; Thomassen and Riisgård, 1995; Turon et al., 1997; Kowalke, 2000; Kumala et al., 2017). These studies related measured clearance rate to pumping rate assuming 100% efficiency of particle retention by the animal’s filter. This technique, although generally employed, may underestimate the volume of water filtered by the animal, since filtration efficiency rarely approaches 100% (Pile et al., 1996; Jimenez, 2011; Morganti et al., 2017). In addition, enclosing a sponge in an incubation chamber causes a reduction in its pumping rate (Parker, 1914; Jørgensen, 1955; Reiswig, 1974; Yahel et al., 2005; Hadas et al., 2008).

More recently dye-tracking techniques (Savarese et al., 1997; Yahel et al., 2005; Tompkins-MacDonald and Leys, 2008; Weisz et al., 2008; Pfannkuchen et al., 2009) and acoustic Doppler velocimeter were used to directly measure the excurrent water velocity *in situ* (Yahel et al., 2003; Leys et al., 2011; McMurray et al., 2014; Lewis and Finelli, 2015; Ludeman et al., 2017). Nevertheless, a comprehensive understanding of pumping behavior *in situ* is still lacking because most of the studies separately examined the effect of environmental factors such as temperature (Frost, 1980; Riisgård et al., 1993), food (Frost, 1980; Stuart and Klumpp, 1984; Huysecom et al., 1988), and suspended sediments (Reiswig, 1971; Gerrodette and Flechsig, 1979; Leys and Lauzon, 1998; Tompkins-MacDonald and Leys, 2008; Strehlow et al., 2016; Grant et al., 2018). In nature, these factors act together, and their effect may vary over the range of natural variation of each factor and its interaction with other factors.

In his pioneering study of sponge pumping rate, Reiswig (1974) observed a reduction in sponge-specific pumping rate as a function of sponge size (i.e., larger specimens pump less water per unit of volume than small specimens). Other reports also provided indications that size might affect sponge pumping (e.g., Riisgård et al., 1993; Thomassen and Riisgård, 1995; McMurray et al., 2014; Lewis and Finelli, 2015; Goldstein et al., 2019). However, this effect was largely ignored (but see, McMurray et al., 2014), and specific pumping rates were commonly averaged to examine aspects such as community pumping rate (Reiswig, 1974; Kahn et al., 2015), sediment resuspension (Gerrodette and Flechsig, 1979), temperature (Riisgård et al., 1993), or the effect of epizoic zoanthids (Lewis and Finelli, 2015).

The aim of this study was to explore the *in situ* variations in the pumping rates of five dominant sponges of the Mediterranean coralligenous community over two annual cycles, covering the full natural range of temperature and other environmental conditions. The Mediterranean Sea is a warm temperate sea, which is characterized by a strong seasonality in terms of temperature and food availability (Ribes et al., 1999b) due to the annual process of stratification of the water column in spring-summer and its homogenization in fall and winter (Coma et al., 2009). The seawater temperature varies about 10° from winter to summer months (from 12–14 to 20–23°C, respectively; Ribes et al., 1999b), and particulate organic matter (POM) concentration exhibits a high inter season variability (from <70 to >300 μg C L^−1^) with maximum peaks of concentration in spring (Ribes et al., 1999b). Therefore, the Mediterranean Sea provides an excellent environmental framework to examine the effects of the seasonal variation of the main environmental factors on physiological parameters such as pumping rate. To elucidate the effect of sponge size on pumping rate, we examined pumping over a range of sponge sizes that reflected the population size distribution at the study area. Our findings suggest that temperature and food concentration had only a minor effect on sponge pumping, while sponge size was found to be the major determinant of the sponge pumping rate.

## Materials and Methods

The study was conducted at the “Parc Natural del Montgrí, les Illes Medes i el Baix Ter” (Northwestern Mediterranean Sea, 42°0.06′N, 3°0.21′E) at 5–10 m depth. *In situ* pumping rates were measured for five Mediterranean sponges every 3-degree interval of natural temperature change over the annual temperature range of 12–23°C, from July 2012 to July 2014. The studied Demospongiae species were *Agelas oroides* (Schmidt, 1864), *Petrosia ficiformis* (Poiret, 1789), *Chondrosia reniformis* (Nardo, 1847), *Crambe crambe*, and *Dysidea avara* (Schmidt, 1862). These species were selected because they represent the most abundant sponge species at the studied area (Teixidó et al., 2013), and they are all common and widespread habitants of the coralligenous community in the Mediterranean Sea (Uriz et al., 1992).

### Environmental Conditions

To measure the availability of particulate food resources for the study sponges (i.e., total particulate organic carbon, POC, and pico- and nanoplankton concentrations), duplicate seawater samples (2 L) were collected by scuba divers in proximity to the sponge community every sampling session.

For pico- and nanoplankton samples, three aliquots of 2 ml were withdrawn and fixed with 1% paraformaldehyde + 0.05% electron microscope grade glutaraldehyde (final concentration, Sigma P6148, and Merck 8.206.031.000, respectively) and placed in ice. Once at the harbor, samples were frozen in liquid nitrogen and stored at −80°C until analysis. *Synechococcus* sp., *Prochlorococcus* sp., autotrophic pico-and nano-eukaryotes, and non-photosynthetic bacteria were quantified with a flow cytometer (FACSCalibur, Becton-Dickinson, 488 nm excitation blue laser) following the method of Gasol and Morán (1999).

For total POC, triplicates of 500 ml were prefiltered through a 100 μm net to remove rare zooplankters and large debris and filtered overs a pre-combusted GF/F glass fiber filter. Filters were then frozen in liquid nitrogen and kept at −80°C until analysis. Prior to the analysis, filters were quickly thawed, folded with vanadium oxide in sterile plaques, fumigated with 37% HCl for 48 h, and dried at 60°C for 24 h. Organic carbon content was determined with C:H:N autoanalyzer (EA 1108 CHNS-O Carlo Erba Instruments).

The carbon content of the pico-and nanoplankton (hereafter live carbon) was then estimated using published conversion factors as follows: *Synechococcus* sp. 470 fg C cell^−1^ (Campbell et al., 1994), eukaryotic algae 1,496 fg C cell^−1^ (Zubkov et al., 1998), and non-photosynthetic bacteria 20 fg C cell^−1^ (Ducklow et al., 1993). Detrital organic carbon (hereafter detrital POC) was estimated as the difference between the total POC measured on filter analysis and the live carbon estimated from cell counts.

The temperature at the study area was continuously recorded using HOBO Pendant (Onset) data loggers that were shaded to avoid heating by direct sunlight.

### Seasonal Pumping Rate

The seasonal study of sponge pumping rate was performed on specimens that were visually healthy and possessed only a single osculum to allow the flow of the entire sponge to be measured from a single excurrent aperture. Care was taken to use similar sized sponges to minimize the size effect on the seasonal pattern (Table 1).

**Table 1:** Mean sponge size (±95% CI) for the single-osculated specimens used for the seasonal study.

Species	*n*	*V* (cm^3^)	*V*_dis_ (ml)	AFDW (g)
*A. oroides*	158	15.88 ± 2.16	6.19 ± 0.84	1.30 ± 0.14
*C. reniformis*	221	16.06 ± 1.46	6.90 ± 0.63	2.48 ± 0.17
*P. ficiformis*	147	7.88 ± 1.26	5.20 ± 0.83	1.04 ± 0.13
*D. avara*	144	3.78 ± 0.60	1.44 ± 0.28	0.04 ± 0.01
*C. crambe*	181	1.20 ± 0.11	0.43 ± 0.04	0.07 ± 0.004

*Data are expressed as in situ volume (V, cm^3^), volume estimated by water displacement (V_dis_, ml), and biomass (ash-free-dry weight, AFDW, g). n, number of sampled specimens*.

Pumping rate was measured using a modification of the dye front speed method (DFS) described by Yahel et al. (2005). Briefly, a transparent tube was positioned as close as possible above the sponge osculum, and the movement of the dye inside the tube was recorded by a diver using a video camera. Unlike Yahel et al. (2005), the dye was not applied inside the tube, instead small amounts of fluorescein dyed seawater were released next to the sponge ostia and inhaled by the sponge just before the sampling.

To ensure minimal interference with animals’ behavior and to avoid deviations from ambient water density, the sodium fluorescein powder was mixed with ambient water drawn into the syringe just before sampling. A disposable syringe filter (25 mm, 0.2 μm) was installed on the syringe to avoid the release of dye particles. A frame-by-frame analysis was used to measure the speed of the dye front inside the tube. The rate of water flow from the osculum was calculated following Yahel et al. (2005) as the product of tube cross-sectional area and the dye front speed, or, in the few cases where the tube was smaller than the osculum, as the product of dye front speed and osculum cross-sectional area. When possible at least 11 specimens from each species were sampled during each sampling session, and three to five replicates were conducted per specimen. Due to the difficulty of sampling during the winter months and the occurrence of the arrest of pumping in some specimens (i.e., the osculum was closed), a minimum of seven specimens were sampled in some cases. To obtain size-specific pumping rates, the average pumping rate of each specimen was then divided by its body size. It should be noted that only sponges that were actively pumping were included in this study.

### Effect of Sponge Size on Pumping Rate

The second series of pumping rate measurements was conducted over a broad size range of multi-osculated sponges to specifically address the nature of the relationship between pumping rate and sponge dimension. These measurements were all performed on the same season (June 2014) under very similar environmental conditions. At least 39 specimens were sampled from each species to estimate the size-specific pumping rate over the range of sponge sizes of each population, which included multi-osculated individuals.

To estimate the size-specific pumping rate for large sponge specimens with multiple oscula, we used the equation

$$PR\;\left(\mathrm{ml}\;\min^{-1}{\left(\mathrm{cm}\;\mathrm{sponge}\right)}^{-3}\right)=\frac{1}{V}\sum_{i}^{n}n_{i}\;\underline{{OFR}_{i}}$$

where *V* is the sponge volume (cm^3^), *i* is the osculum size categories (see below), *n_i_* is the number of oscula from the *i*th osculum size category in the studied specimen, and $\underline{{OFR}_{i}}$is the mean oscular pumping rate (ml min^−1^) of the *i*th osculum size category. For this analysis, the oscula were divided into three size categories within each sponge species, as a function of the maximum osculum diameter of that species. For *D. avara*, *C. crambe*, and *A. oroides*, these size classes were small ≤ 2 mm, 2 < medium ≤4 mm, large >4 mm. For *P. ficiformis*, these size classes were small ≤ 2 mm, 2 < medium ≤ 3 mm, large > 3 mm. For *C. reniformis*, the classes were small ≤ 2.5 mm, 2.5 < medium ≤ 5 mm, large > 5 mm.

Three individuals, two *C. crambe* (sponge volume > 30 cm^3^) and one *D. avara* (sponge volume > 400 cm^3^) specimens, were removed from this analysis because they were out of the population size range.

### Pumping Rate Method Comparison

Before commencing the seasonal sampling, we performed a cross calibration between the modified dye speed method (DS, this study) and the original dye front speed method (DFS; Yahel et al., 2005). Only specimens with one osculum were selected to allow the flow of the entire sponge to be measured from a single excurrent aperture. At least 17 different specimens, haphazardly selected, were tested for each sponge species with a minimum of three replicates per each method employed. To test if the estimated pumping rate significantly differed between the two methods, a paired *t* test was used over a log or square root transformed data to satisfy the normality and/or heteroscedasticity assumptions. Our results indicated that both methods produced nearly identical results for all five examined sponges (*t* test, *p* > 0.05) from a minimal estimated deviation of 3% for *C. reniformis* up to maximal deviation of 32% for *C. cramb*e (Supplementary Table S1).

### Determination of Sponge Size

To estimate the sponge size and oscula dimensions, each sponge specimen and osculum sampled were photographed close to a ruler and subsequently measured using manual delineation with Image J (Ver. 64). Sponge height and dimensions were measured in the field. Sponge volume was calculated by multiplying the area with height, based on the approximated single or combined geometrical shapes that characterized each specimen. Estimates of sponge volume generated by this method closely resembled estimates made by 3D video analysis (Moskovitch, Diga, and Yahel, unpublished data).

The relations between sponge volume measured *in situ* and volume by water displacement, sponge dry weight, and sponge biomass (ash-free dry weight) were determined in order to relate these variables of body size with the sponge pumping rate measurements. At least 30 individuals of each species were measured *in situ* as described above and then collected, and their volume was measured again in the laboratory. The sizes of each individual were measured and expressed in several ways: volume by water displacement (*V*_dis_, ml of displaced water in a graduated cylinder); dry weight (DW, 100°C, 24 h); and ash-free dry weight (AFDW). The ash-free dry weight (AFDW, g) was calculated by subtracting the ash weight (after 6 h combustion of the DW samples in a muffle furnace at 500°C) from the sponge dry weight.

### Statistical Analyses

To estimate the minimum sample size needed to examine pumping rate, we used the standard error (SE) sample size method (Supplementary Figure S1). A total of 54 specimens of *C. reniformis* were haphazardly selected, their pumping rate was measured as described above, and the recorded values were recombined into random groups of increasing number from 1 to 18, and the SE of each group was plotted against sample size (Supplementary Figure S1). SE quickly stabilized at a sample size of about seven, where it became approximately 5–10% of the mean.

Multiple regression analysis was used to establish the contribution of different environmental and allometric variables to the observed variation in pumping rates within each species. Variables were square root or ln-transformed to satisfy the normality and/or heteroscedasticity assumptions, and a complete residuals analysis was performed to validate the robustness of the resulting model.

The metabolic response coefficient for temperature variations, Q_10_, was estimated by applying Van’t Hoff’s formula (Clarke, 1983):

$$Q_{10}={\left(\frac{{PR}_{T2}}{{PR}_{T1}}\right)}^{\frac{10}{T_{2}-T_{1}}}$$

where PR_T1_ and PR_T2_ are the mass-specific pumping rates (ml min^−1^ g AFDW^−1^) at temperatures *T*_1_ and *T*_2_ (°C), respectively.

To ensure that the sampled sponge size is comparable (within each species) throughout the study period, ANOVA was run to detect significant differences between the sponge size ranges at different sampling sessions. The results showed that sponge sizes were significantly higher in the first sampling session than all other sessions in *C. crambe* and *D. avara* (ANOVA, *F*_(10, 170)_ = 5.71, *p* < 0.001 in *C. crambe* and *F*_(10, 133)_ = 4.77, *p* < 0.001 in *D. avara*). Therefore, these measurements were removed from the statistical analysis.

Because water viscosity has been suggested to be a major controller for the pumping rate of suspension feeders (Riisgård and Larsen, 2010), the kinematic viscosity of the water was calculated as $v\;\left(10^{-6}m^{2}s^{-1}\right)=0.0005^{\frac{2260}{K}}\text{,}$ where K is the water temperature in Kelvin (Larsen and Riisgård, 2009). The expected volume-specific pumping rate for each sponge at each viscosity level was calculated using the equation PR_v_ = *aV*^−2^ (Larsen and Riisgård, 2009) using the pumping rate measured at the lowermost temperature (13°C) as a starting point (coefficient *a*) and an exponent of −2 (the average of all *b* values reported in Table 1 of Larsen and Riisgård, 2009).

One-way ANCOVA was conducted to test for differences in the slope of the regression of volume-specific pumping rate over the sponge size between the studied species. The difference between the intercepts was tested by building successive models using different species as the reference group.

Statistical analyses were performed with R studio (version 3.2.1) and plotted with SigmaPlot 12.5. Power analysis was performed with G*Power (Ver. 3.194).

## Results

### Environmental Conditions

**Figure 1:**
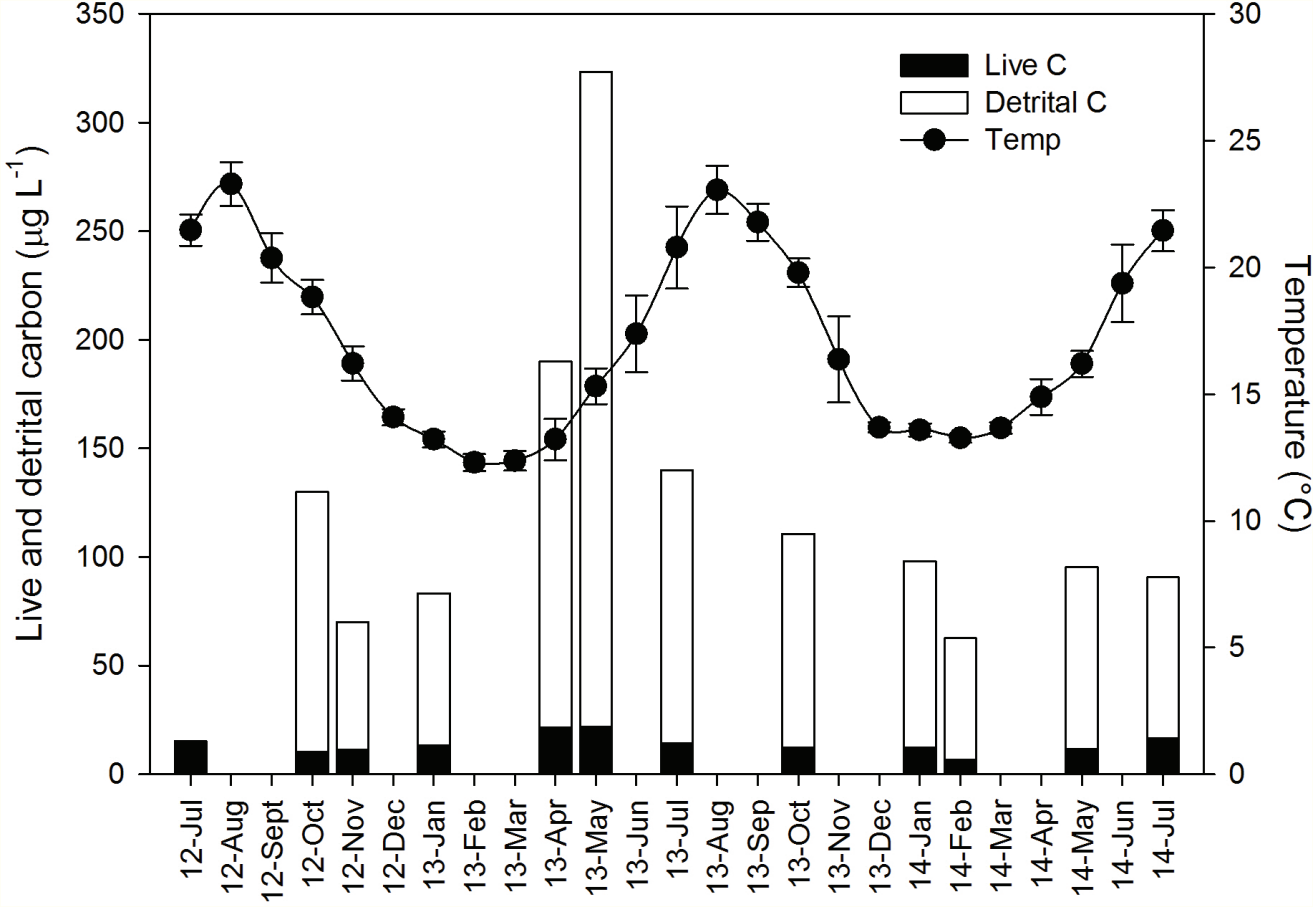
Ambient conditions and its seasonal variation during the sampling period (July 2012–July 2014) in the water column of the study area. Black dots represent the mean temperature (±SD). Particulate organic carbon concentration (μg C L^−1^) is presented as stack bars composed of live cells (black) and detrital carbon (empty). Detrital organic carbon was not quantified in the first sampling (July 2012).

Seawater temperature at 5 m depth exhibited a seasonal pattern, with minimal values (12–14°C) from December to March and maximal values (20–23°C) during the summer months from June to September (Figure 1). Mean annual temperature did not vary among the sampling years (mean ± SD: 17.18 ± 0.81°C).

Detrital organic carbon (detrital C, μg L^−1^) represented the main organic carbon fraction among the potential particulate food resources available for the sponges at the study site and its concentrations were about one order of magnitude higher than the live POC (Figure 1). Suspended detrital organic C exhibited a marked seasonal pattern with high values in spring and low levels in winter (Figure 1). Non-photosynthetic bacteria also exhibited a marked seasonal pattern with high values in spring and early summer and a rapid decrease toward the autumn. The non-photosynthetic bacteria were the most abundant cell type throughout the sampling period with a mean (±SD) of 2.25 × 10^5^ ± 0.90 × 10^5^ cell ml^−1^ and a maximum concentration of 4.39 × 10^5^ ± 0.46 × 10^5^ cell ml^−1^ (registered in May 2013). *Synechococcus* sp. was the second most abundant group (1.45 × 10^4^ ± 0.69 × 10^4^ cell ml^−1^) and, similar to non-photosynthetic bacteria, was more abundant in spring-summer, with lower values in winter. *Prochlorococcus* sp. concentrations were very low (3.45 × 10^3^ ± 3.83 × 10^3^ cell ml^−1^) and exhibited a different seasonal pattern, with high values in autumn-winter and almost absence in spring-summer. It should be noted that the quantification of *Prochlorococcus* in surface water is problematic, and some of these cells were likely counted as non-photosynthetic bacteria (Dusenberry and Frankel, 1994; Collier, 2000). Autotrophic pico-and nanoplankton represented the lowest fraction in terms of abundances among pico-and nanoplankton communities (1.6 × 10^3^ ± 1.04 × 10^3^ cell ml^−1^). Pico-eukaryotes (1.27 × 10^3^ ± 1.00 × 10^3^ cell ml^−1^) followed a marked seasonal pattern, with high values in winter and low levels in summer. By contrast, nano-eukaryotes (3.83 × 10^2^ ± 2.83 × 10^2^ cell ml^−1^) exhibited little seasonal variation, with only one peak observed in spring 2013.

### Sponge Size

Field-based measurements of sponge area and volume provided a good approximation of the actual sponge volume and biomass (Table 2). For example, sponge volume estimated from sponge dimensions measured *in situ* was linearly correlated with their actual volume measured by water displacement at the laboratory (*R*^2^ > 0.7; Table 2). Water displacement volume was always lower due to the perforated nature of the sponge body (slope < 1; Table 2), and this effect was more prominent for the LMA sponges for which water displacement volume was <36% of the total volume. For the HMA sponges, the relationship between *in situ* measured volume and water displacement volume ranged from 39% for *A. oroides* to 66% for *P. ficiformis.*

**Table 2:** Relationship between size parameters measured *in situ*: volume (by fitting geometric shapes, cm^3^) and area (cm^2^); and in the laboratory: dry weight (DW, g), ash-free dry weight (AFDW, g), and volume measured by water displacement (*V*_dis_, ml).

Measured in the lab	Measured *in situ*	*C. reniformis* (*n* = 40)			*A. oroides* (*n* = 30)			*P. ficiformis* (*n* = 30)			*C. crambe* (*n* = 31)			*D. avara* (*n* = 30)			Function
		*a*	*b*	*R*^2^	*a*	*b*	*R*^2^	*a*	*b*	*R*^2^	*a*	*b*	*R*^2^	*a*	*b*	*R*^2^	
DW (g)	Area (cm^2^)	0.36	0.87	0.87	0.57	0.81	0.81	0.86	0.86	0.89	0.03	0.70	0.84	0.01	1. 35	0.92	*Y* = *ax^b^*
	Volume (cm^3^)	0.41	0.75	0.85	0.22	0.79	0.87	0.74	0.78	0.93	0.09	0.70	0.84	0.01	1. 35	0.92	
AFDW (g)	Area (cm^2^)	0.27	0.89	0.86	0.41	0.79	0.81	0.29	0.84	0.88	0.02	0.70	0.84	0.005	1.35	0.93	
	Volume (cm^3^)	0.31	0.78	0.84	0.15	0.79	0.89	0.22	0.78	0.94	0.062	0.70	0.84	0.004	1.35	0.93	
*V*_dis_ (ml)	Volume (cm^3^)	0.00	0.43	0.71	0.00	0.39	0.81	0.00	0.66	0.88	0.00	0.36	0.82	0.00	0.33	0.90	*Y* = *bx* (*a* = 0)

*The coefficients of the regression analysis function that best describes this relationship (a and b) as well as the R^2^ are provided for each sponge. The function used (linear or power) is provided in the right column. All regressions were highly significant (the probability that b is zero was <0.001 in all cases)*.

Sponge area and volume measured independently *in situ* were also closely related to the sponge dry weight and biomass (ash-free dry weight), and these relationships were best described by a power function yielding *R*^2^ ≥ 0.81 (Table 2). As reflected in the intercepts of the power function relating to sponge biomass and volume, the biomass density of the HMA sponges was 2–77 higher than LMA sponges (Table 2).

The average sizes (mean ± 95% CI) of the single-osculated specimens that we used for the seasonal sampling study ranged from 1.2 ± 0.1 cm^3^ for *C. crambe* to 16.1 ± 1.5 cm^3^ for *C. reniformis* (Table 1). Sponges used for the size effect study ranged in size from 0.8 to 440 cm^3^ (Table 3).

**Table 3:** Allometric function *PR = aV^b^* between sponge pumping rate (PR, ml min^−1^) and its size (*V*, cm^3^) calculated for multi-osculated specimens.

Species	*n*	*a*	*b*	*R*^2^	*p*	Volume range (cm^3^)
*D. avara*	39	44.83 ± 16.27	0.50 ± 0.09	0.54	<0.0001	1.5–148
*C. crambe*	39	23.88 ± 5.65	0.77 ± 0.09	0.74	<0.0001	0.8–25
*P. ficiformis*	40	10.41 ± 4.48	0.65 ± 0.08	0.74	<0.0001	3–420
*C. reniformis*	41	18.03 ± 7.42	0.61 ± 0.09	0.69	<0.0001	3–180
*A. oroides*	40	18.52 ± 6.93	0.46 ± 0.07	0.64	<0.0001	3–440

*Data are expressed as the regression coefficient ± standard error. n is the number of specimens sampled. The probability that the exponent b is zero was <0.0001 for all species*.

### Seasonal Pumping Rates

**Figure 2:**
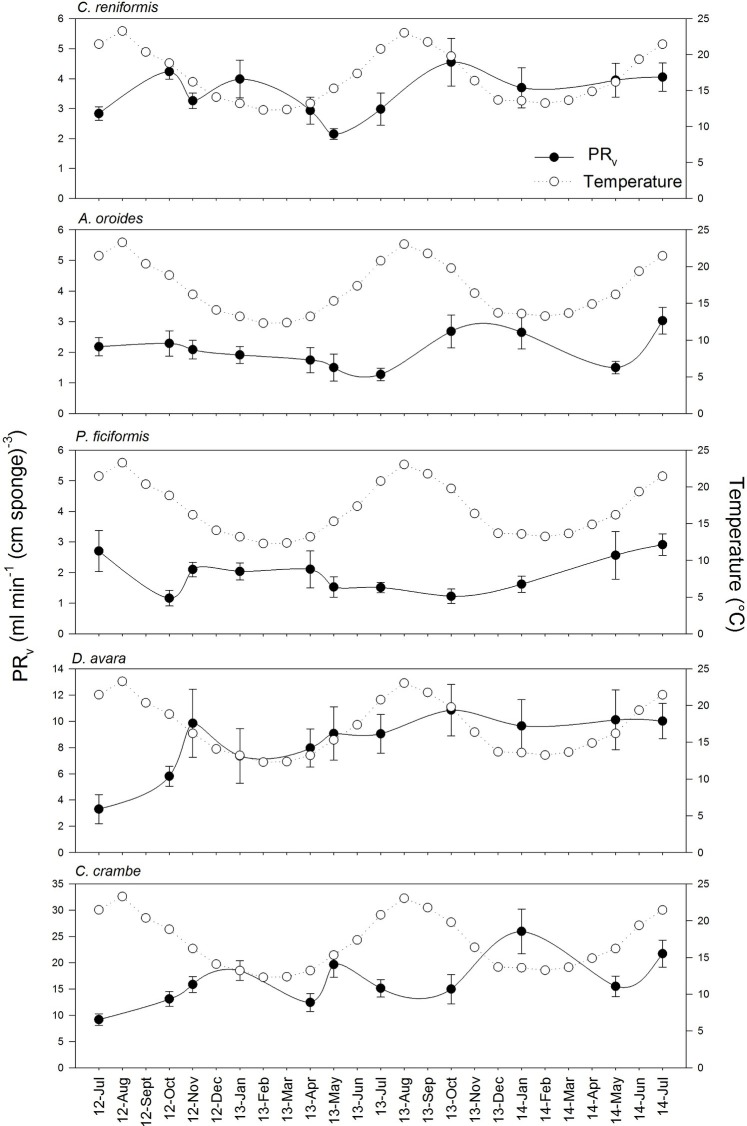
Variation of volume-specific pumping rates [PR*_V_*, ml min^−1^ (cm sponge)^−3^] throughout the years in the five study species. The temperature variation over the annual cycles at the study site is included in each species graph as empty dots. Data are expressed as mean ± SE. The maximal seasonal variation of PR*_V_* was 2.5 folds, depending on the species: *A. oroides*, 2.4; *C. reniformis*, 2.1; *P. ficiformis*, 2.5; *D. avara*, 1.9; *C. crambe*, 2.1.

Volume-specific pumping rate varied seasonally by 2 to 2.5 folds depending on the species, but no clear seasonal trend was observed for pumping rate (Figure 2; Supplementary Table S2). Our *in situ* measurements and subsequent multiple regression analysis suggest that temperature and particulate food concentration had either weak or non-significant control on pumping rate (Table 4). Pumping rates were negatively correlated with the concentration of detrital organic particles in all five species, but this trend was statistically significant only for *P. ficiformis* and *A. oroides.* Pico-and nanoplankton concentration was significantly correlated only with the pumping rate of *P. ficiformis* (Table 4).

**Table 4:** Multiple regression analysis to estimate the effect of temperature (°C), sponge size (ln-volume, cm^3^), and the concentrations of pico-and nanoplankton cells (Live C, μg L^−1^), and detrital particulate organic carbon (Detrital POC, μg L^−1^) on the volume-specific pumping rate [PR*_V_*, ml min^−1^ (cm sponge)^−3^] of single-osculated sponge specimens with similar size.

Variable	*A. oroides*	*C. reniformis*	*P. ficiformis*	*D. avara*	*C. crambe*
Temperature (°C)	0.19***	0.09	0.13	−0.02	−0.01
ln-volume (cm^3^)	−0.79***	−0.66***	−0.62***	−0.80***	−0.59***
Live C (μg L^−1^)	0.06	−0.07	0.26*	−0.03	−0.02
Detrital POC (μg L^−1^)	−0.26***	−0.11	−0.42***	−0.04	−0.08
a (ml min^−1^ cm^−3^)	1.69 ± 0.29***	2.41 ± 0.26***	0.55 ± 0.33	2.83 ± 0.32***	2.81 ± 0.23***
*R*^2^	0.70***	0.50***	0.45***	0.63***	0.36***

*Values are the standardized regression coefficients for each variable (rows) and each species (columns). The Intercept (a) and R squared (R^2^) are also presented below. The first sampling session (July 2012) for C. crambe and D. avara was not included in this analysis because the sponge size range was significantly higher than other sampling periods. Bold values are statistically significant*.

*** p < 0.001;

** p < 0.01;

* *p < 0.05*.

**Figure 3:**
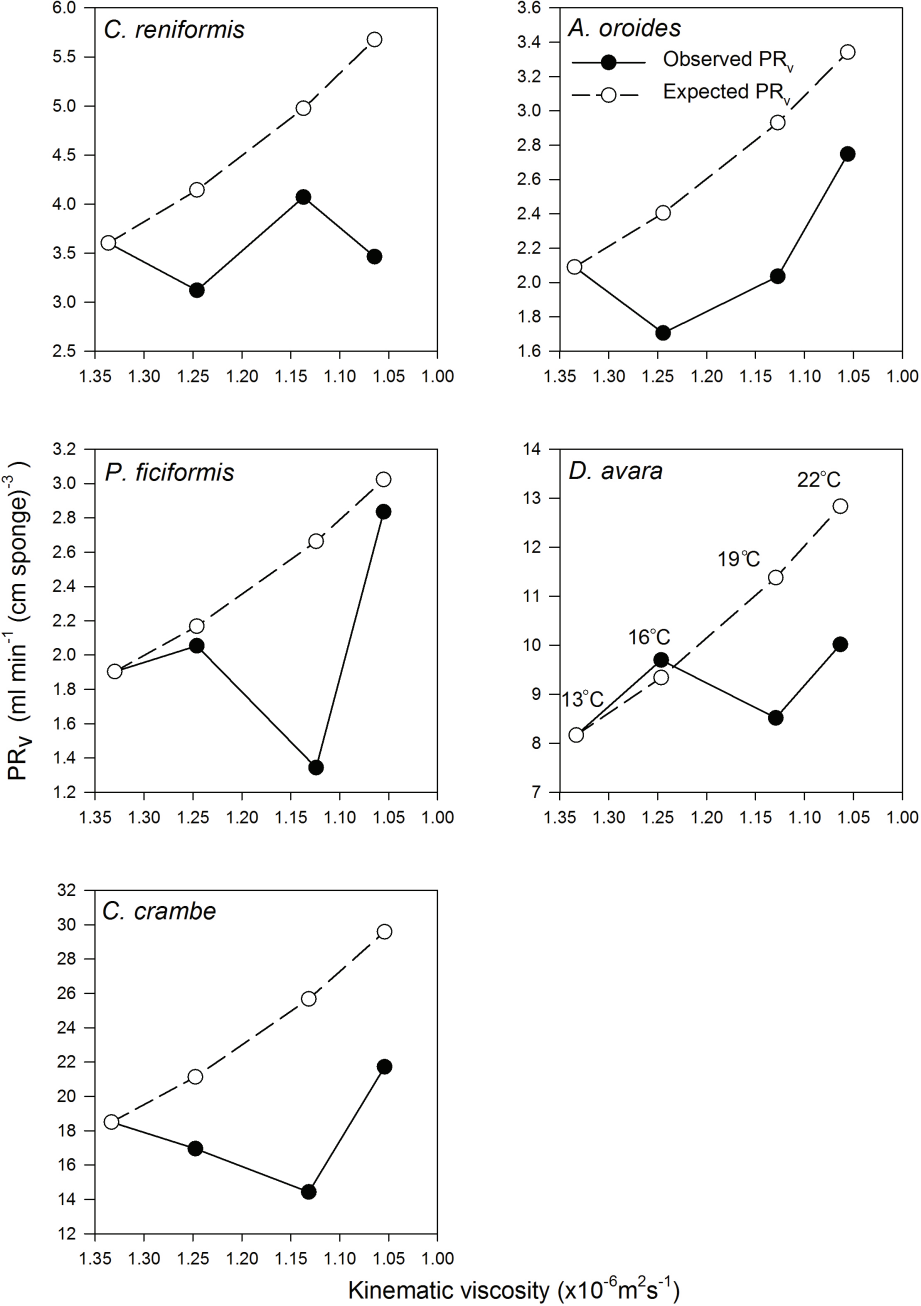
The relationships between volume-specific pumping rate [PR*_V_*, ml min^−1^ (cm sponge)^−3^] and the kinematic viscosity (*v*, ×10^−6^ m^2^ s^−1^) in the five study species examined seasonally. Observed values are presented as full circles. Expected values are presented as empty circles (see Methods text for details). Error bars were omitted for the clarity of the representation. Temperatures are indicated at different viscosities in the *D. avara* panel but pertain to all panels.

Surprisingly, the temperature had very little effect on sponge pumping rate (Table 4, Figures 2, 3). Temperature was significantly correlated only with the pumping rate of *A. oroides*, but for all five species, *in situ* pumping rate did not increase monotonically with temperature over the temperature range at the study site (from 12 to 23°C; Figures 2, 3), and Q_10_ was low (ranging from 1 to 1.98) for all the studied species. As shown in Figure 3, the predicted effect of an increase in pumping rate as viscosity decrease due to its temperature-dependence did not match with the observed data. At 22°C, the observed pumping rates were about 1.5-fold lower than expected in *C. reniformis* and *D. avara*, and a similar mismatch was observed at 19°C for *A. oroides*, *P. ficiformis*, and *C. crambe* (Figure 3).

In contrast to the weak effect of environmental parameters, all five species exhibited a clear and significant pattern of decrease in volume-specific pumping rate with sponge size (multiple regression analysis, *p* < 0.001; Table 4). Despite our effort to focus on similar size (small single-osculated sponge specimens; Table 1), size had the largest and most significant effect on the pumping rates of all five species during the seasonal study both within each sponge species (e.g., Table 4) and among species.

### Pumping Rate and Sponge Size

In order to properly determine the nature of the size-dependent pumping rate relationship, a second series of pumping rate measurements was conducted on specimens representative of the entire size range distribution of each species at the study site. As many of the larger sponges possessed more than one osculum, the pumping rate of each specimen (PR, ml min^−1^) was calculated as the sum of the pumping rates of all oscula within that specimen.

**Figure 4:**
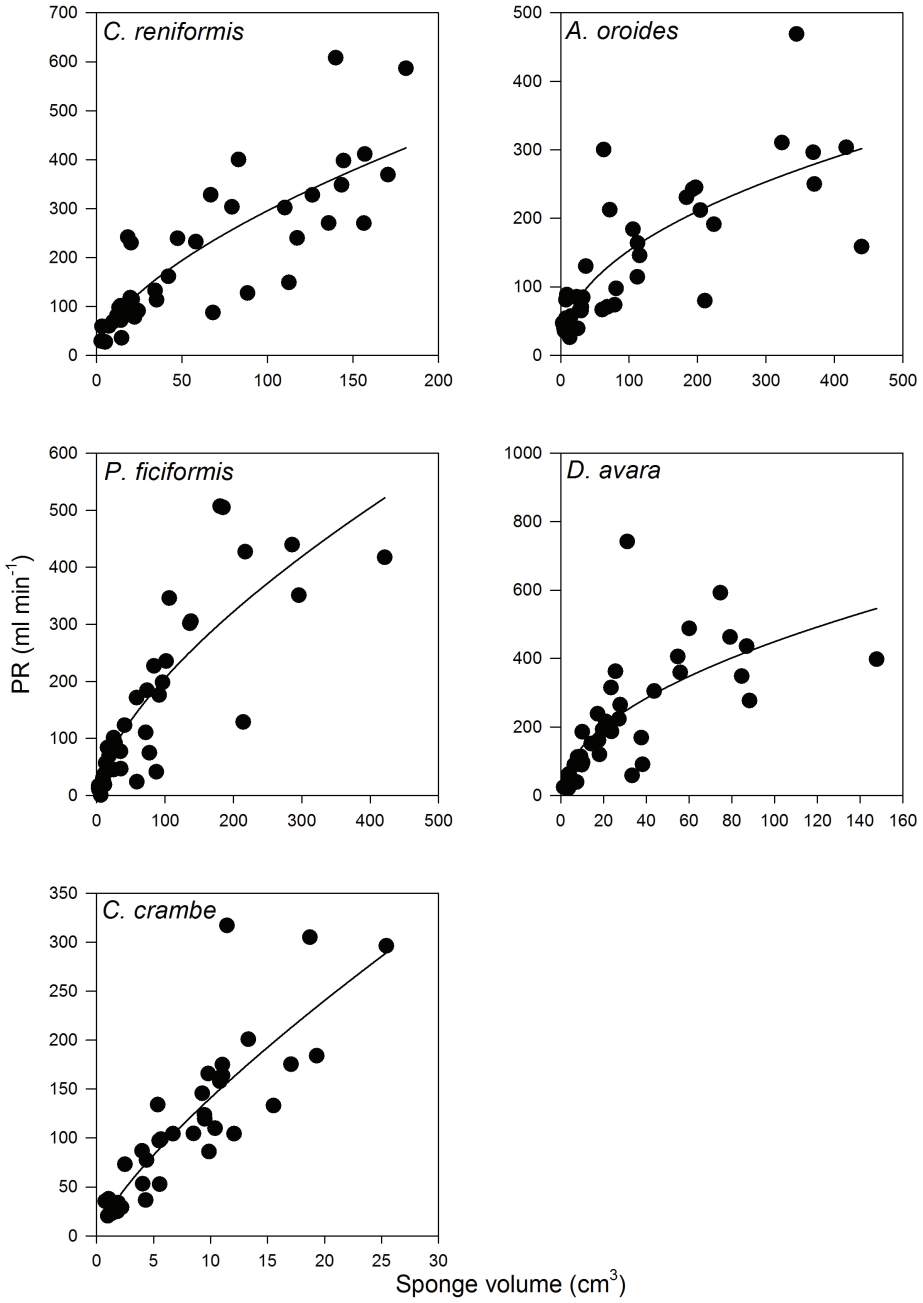
The relationships between the sponge pumping rate (PR, ml min^−1^) and its volume (cm^3^) for multi-osculated specimens. The equations of the regression lines are shown in Table 3.

For all five species, pumping rate increased with increasing of sponge size and these relationships were best described by the allometric function (PR *= aV^b^*) with exponents ranging from 0.46 to 0.77 (Figure 4, Table 3). The allometric increase of pumping rate with sponge size reported above, as a function of sponge volume, was similar to that observed using biomass (AFDW, g; Supplementary Figure S2). In the following text, we use mostly volume as a descriptor of size because it can be estimated with non-destructive methods, and it is the most commonly used descriptor of size in the literature.

**Figure 5:**
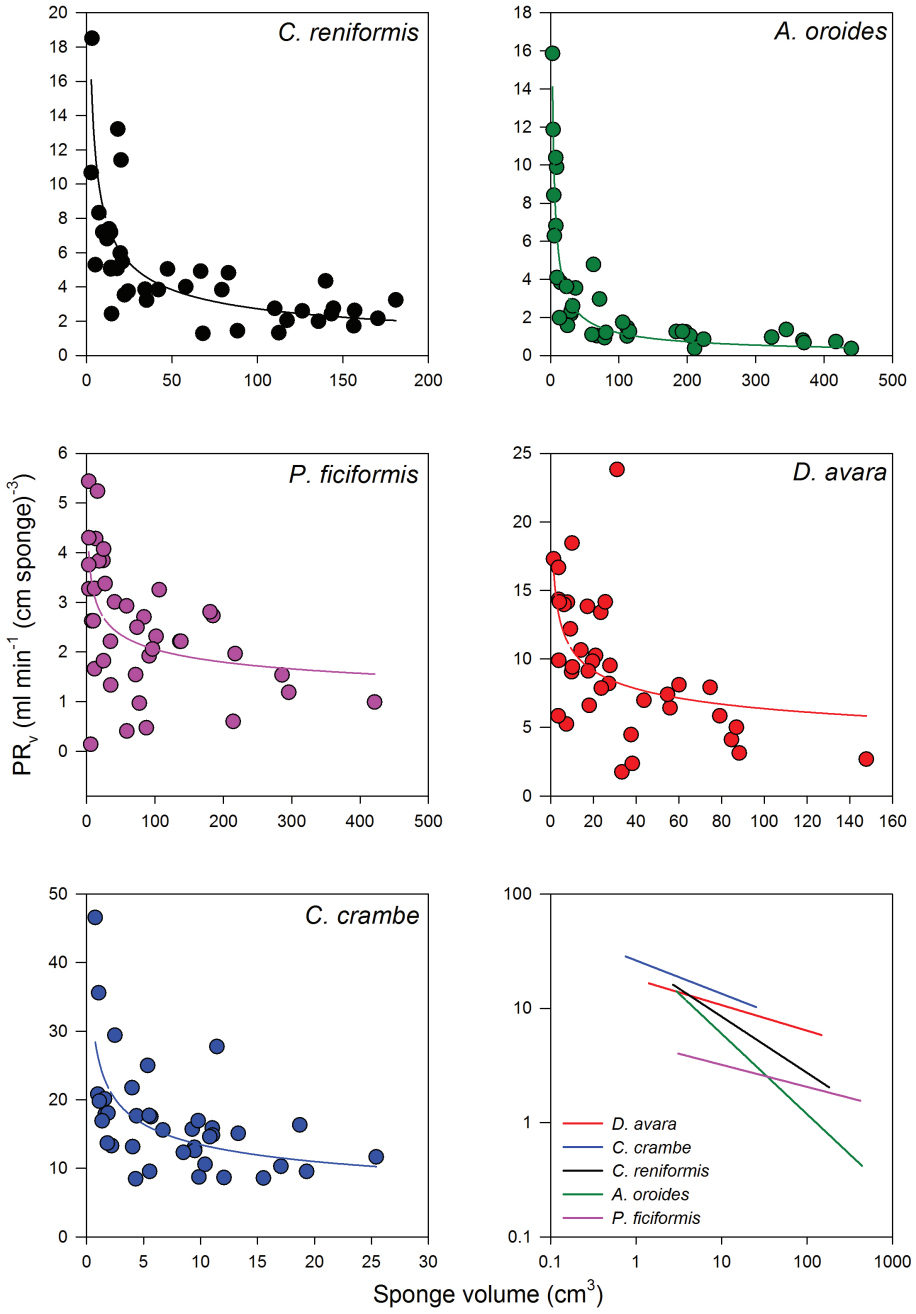
The relationships between the volume-specific pumping rate [PR*_V_*, ml min^−1^ (cm sponge)^−3^] and sponge volume (cm^3^) on multi-osculated specimens. The regression lines of all five species are plotted together on a log scale in the bottom right panel. The equations of the regression lines are shown in Table 5.

Within the natural size distribution of each species, the volume-specific pumping rate [PR*_V_*, ml min^−1^ (cm sponge)^−3^] varied from 3 to 33 folds (Figure 5, Table 5). In all five species, volume-specific pumping rate decreased with the increase in sponge volume (cm^3^), conforming to a power function with negative exponents ranging from −0.2 to −0.7 (Figure 5, Table 5).

**Table 5:** Allometric function PR*_V_ = aV^b^* between volume-specific pumping rate [PR*_V_*, ml min^−1^ (cm sponge)^−3^] and sponge size (*V*, cm^3^) calculated for the same specimens as in Table 3 above.

Species	*a*	*b*	*R*^2^	*p*	Sponge size (cm^3^)		Estimated PR*_V_* [ml min^−1^ (cm sponge)^−3^] for		PR*_V_* ratio
					*V*_max_	*V*_min_	PR_*V*max_	PR_*V*min_	*V*_min_/*V*_max_
*D. avara*	17.88 ± 2.94	−0.22 ± 0.06	0.28	0.0007	148	1.5	6	17	3
*C. crambe*	26.24 ± 2.23	−0.29 ± 0.06	0.38	<0.0001	25	0.8	10	28	3
*P. ficiformis*	5.01 ± 0.71	−0.19 ± 0.04	0.34	<0.0001	421	3.12	1.6	4	3
*C. reniformis*	26.31 ± 4.84	−0.49 ± 0.07	0.54	<0.0001	181	2.72	2	16	8
*A. oroides*	30.39 ± 3.56	−0.70 ± 0.06	0.86	<0.0001	440	2.98	0.4	14	33

*Data are expressed as the regression coefficient ± standard error. To illustrate the potential range of such estimates V_max_ and V_min_ are the volumes of the largest and smallest multi-osculated specimens measured for each species, and PR_Vmax_ and PR_Vmin_ are the estimated volume-specific pumping rates calculated for these specimens using the allometric functions reported in this table. The ration of these estimated PR_V_ varied from 3 to 33 folds depending on species. The probability that the exponent b is zero was <0.001 for all species*.

The high variability and the strong dependency of the volume-specific pumping rate of the sponges on their size determine that the traditional comparison of mean volume-specific pumping rate among species is irrelevant. Therefore, we used the parameters of the allometric function of PR*_V_* over size (Table 5) to compare pumping rate characteristics among the examined species. A one-way ANCOVA, run using different species as a reference group, showed that *P. ficiformis* had a statistically lower intercept [mean ± SE, 5.01 ± 0.71 ml min^−1^ (cm sponge)^−3^] than the other species. The other four species exhibited statistically similar high intercepts [mean ± SE: 25.20 ± 2.63 ml min^−1^ (cm sponge)^−3^] suggesting that very small sponges (volume of 1 cm^3^) from different species have high and very similar volume-specific basal pumping rates. The slopes of volume-specific pumping rate over sponge size (i.e., the exponent or the rate of decline) varied significantly among species (one-way ANCOVA, *F*_(4, 189)_ = 47.97, *p* < 0.001; Table 6). Exponents ranged from −0.7 for *A. oroides* and −0.5 for *C. reniformis* to ~−0.2 for the other three species (Table 5, Figure 5). The more negative exponents of *C. reniformis* and *A. oroides* resulted in larger variation of the volume-specific pumping rate for these species (8 and 33 folds, respectively, see Table 5), while the less negative exponents in *P. ficiformis*, *D. avara*, and *C. crambe* resulted in smaller variation of the volume-specific pumping rate (~3 folds; Table 5).

**Table 6:** A pairwise comparison (Tukey test) of the slopes of the allometric function PR*_V_ = aV^b^* (Table 5).

*p* values	*D. avara*	*C. crambe*	*C. reniformis*	*A. oroides*	*P. ficiformis*
*D. avara* (−0.22 ± 0.06)	—				
*C. crambe* (−0.29 ± 0.06)	0.9796	—			
*C. reniformis* (−0.49 ± 0.07)	0.4959	0.2356	—		
*A. oroides* (−0.70 ± 0.06)	**0.0060**	**0.0019**	0.3409	—	
*P. ficiformis* (−0.19 ± 0.04)	0.8883	0.9995	**0.0450**	**<0.0001**	—

*PR_V_ is the volume-specific pumping rate, and V is the volume of the multi-osculated sponge specimens sampled. The slopes ± SE (b) are reported in brackets for each sponge species on the left column. TP values represent the probability that two slopes are different. Significant differences (p < 0.05) are bolded*.

## Discussion

While our knowledge of sponge pumping has been greatly expanded in recent years, the pioneering work of Reiswig (1971, 1974), who described the natural variation of sponge pumping rate of Caribbean species over a long period of time (days to months), remains to date the most comprehensive study of *in situ* sponge pumping. Reiswig found that sponge-pumping behavior was more complex than previously thought and might be driven by both abiotic (e.g., storms, sedimentation, and temperature) and intrinsic factors (e.g., daily cycles and behavioral pattern). More recent studies focused on the effect of a single parameter on the sponge pumping in either laboratory conditions (e.g., Annandale, 1907; Stuart and Klumpp, 1984; Huysecom et al., 1988; Riisgård et al., 1993; Larsen and Riisgård, 1994; Tompkins-MacDonald and Leys, 2008; Pfannkuchen et al., 2009; Schläppy et al., 2010; Massaro et al., 2012; Lavy et al., 2016; Strehlow et al., 2016; Kumala et al., 2017; Kumala and Canfield, 2018; Goldstein et al., 2019) or *in situ* (e.g., Gerrodette and Flechsig, 1979; Savarese et al., 1997; Bell et al., 1999; Trussell et al., 2006; Tompkins-MacDonald and Leys, 2008; Schläppy et al., 2010; Leys et al., 2011; McMurray et al., 2014; Lewis and Finelli, 2015; Ludeman et al., 2017; Grant et al., 2019; Wooster et al., 2019). Unfortunately, most studies were short term or small scale and thereby failed to provide a comprehensive view of the long-term *in situ* natural pumping behavior of the studied sponges. In this study, we used a dye technique to estimate the volume of water processed by 10 specimens from each of five of the most prominent sponge species of the coralligenous community in the temperate NW Mediterranean Sea every 3-degree of natural temperature change over two annual cycles. Our *in situ* measurements of sponge pumping rate provided a comprehensive picture of sponge pumping activity under the full range of natural conditions to which the organisms were subjected to over the year and allowed us to evaluate the relative importance of the main factors affecting sponge pumping activity.

The synergistic effect of several factors acting together can confound the effect of any single factor studied in isolation. Therefore, our *in situ* study cannot replace controlled physiological experiments aimed to elucidate the distinct effect of different environmental parameters on sponge pumping rate. Instead, we provide a complementary eco-physiological approach that compares the effect of different factors such as resource composition and abundance, natural temperature variation, the seasonal pattern of gametogenesis, and sponge size as they control the sponge performance in its natural settings.

Our method was limited to daylight and calm sea conditions. Moreover, only specimens that showed fully open oscula were sampled. Consequently, temporal effects such as contraction or expansion of osculum (Reiswig, 1971; Kumala et al., 2017; Goldstein et al., 2019) and day-night cycle (Reiswig, 1971; McMurray et al., 2014; Strehlow et al., 2016) were not accounted in this study. In few cases (0–4% depending on the species), the cross-sectional area of the tube used to measure the dye front speed was more than 40% larger than the osculum cross-sectional area. In these cases, mostly associated with small specimens, the sponge pumping rate might have been underestimated (Yahel et al., 2005).

### Sponge Size Is the Major Determinant of Pumping Rate

In all five demosponge species studied (two LMA and three HMA sponges), sponge size explained most of the observed variance in specific (normalized to mass or volume) pumping rate measured during the ambient seasonal conditions prevailing at the temperate oligotrophic Mediterranean Sea (Table 4). Volume-specific pumping rate [PR*_V_*, ml min^−1^ (cm sponge)^−3^] decreased with the increase of sponge size (V, cm^3^) following the allometric power function PR*_V_ = aV^b^* with negative exponents ranging from −0.2 to −0.7 (Figure 5, Table 5). This observation contrasts previous suggestions that isometry rather than allometry should be expected in sponges due to their modular construction (Riisgård et al., 1993; Thomassen and Riisgård, 1995; McMurray et al., 2014; Lewis and Finelli, 2015). However, allometric scaling has also been observed in modular organisms such as bryozoans (White and Kearney, 2014; Barneche et al., 2016; Burgess et al., 2017).

Empirical data from the literature also supported a volume-specific allometric power function with a negative exponent for sponges (*b* < 0, *Aplysina fistularis*, Reiswig, 1981; *Halichondria panicea*, Riisgård et al., 1993; *Dysidea avara*, Ribes et al., 1999a; *Mycale acerata* and *Isodictya kerguelensis*, Kowalke, 2000). Reiswig (1974) also commented on the high activity of small sponge individuals and the decrease in the specific pumping rate in larger specimens. A decrease in the specific pumping rate with size was also reported for large specimens of the giant barrel sponge *Xestospongia muta* (McMurray et al., 2014).

General models for metabolic scaling (Enquist et al., 2003; Brown et al., 2004) predicted an allometric function in which the exponent (*b*) for mass-specific metabolic rates was about −1/4 (but see White et al., 2007), consistent with the measured exponent for three of the five species we studied (Table 5). However, the rate of reduction of volume-specific pumping rate with the increase in size was twice larger than expected for *C. reniformis* (*b* = −0.49 ± 0.07) and almost three times larger for *A. oroides* (*b* = −0.70 ± 0.06). These results are consistent with previously observed values in *Halichondria panicea* (−0.87) and *Haliclona urceolus* (−0.39) (recalculated from Tables 1, 2 in Riisgård et al., 1993) and *D. avara* (*b* < −1) (Ribes et al., 1999a).

A reduction in size-specific pumping rate with sponge size was attributed to factors such as reduced surface-volume ratio (Frost, 1980), fewer living choanocytes per unit of colony volume in larger sponges (Riisgård et al., 1993; Kowalke, 2000), and other physiological size limits (Reiswig, 1974; Riisgård et al., 1993). It has also been related to the greater age of some big individuals (McMurray et al., 2014). The allometric dependence of specific pumping rate on the size in *C. crambe*, a species with incrusting morphology, is not consistent with the hypothesis of a reduction in the surface to volume ratio with an increase in sponge size. Therefore, the hypothesis of a decrease in the proportion of choanocyte chambers with an increase in sponge size is better supported by the data as well as by the positive correlation between pumping rates and choanocyte chamber density reported by Massaro et al. (2012).

In larger demosponges, conditions such as oxygen concentration (Hoffmann et al., 2008) and metabolic activity of the associated microbes (Subina et al., 2018) are more likely to change in different sections inside the sponge. Moreover, the unidirectional fluid transport system and longer flow path (Reiswig, 1975) might result in a higher head loss (Reiswig, 1975; Riisgård et al., 1993) and a depletion of food and oxygen in the water reaching the deeper choanocytes. Such effects, along with potential enrichment with excretion products in these waters, are likely to result in deeper chambers being less oxygenated, having poor particle supply, and being enriched with metabolites. Such conditions might lead to asynchronous choanocyte activity, with the deeper ones likely less active. Indeed, the metabolic activity of the associated microbes, as well as oxygen concentration inside sponges, change in different sections of sponge tissue (Hoffmann et al., 2008; Lavy et al., 2016; Subina et al., 2018).

The low seasonal effect, low dependency on environmental factors, and the strong size dependence of specific pumping rate of the examined species suggest that the sponge pumping rates may be reliably estimated (±50%) by measuring the sponge size and provided that the constants of the allometric function have been determined. If sponge pumping rate can be reliably approximated using its size, this information can boost the quantification of *in situ* feeding and respiration rates and be used for estimates of the magnitude of sponge-mediated energy and nutrient fluxes at the community level. It is, therefore, crucial to examine whether and to what extent our observations can be corroborated and generalized to other sponge species and habitats, preferably using complementary methodology.

The productivity of the warm temperate Mediterranean Sea (0.59 g C m^−2^ d^−1^) is low in comparison to that of cold temperate areas such as the NW and NE Atlantic shelves (1.48 and 2.00 g C m^−2^ d^−1^, respectively) and the California upwelling (1.06 g C m^−2^ d^−1^), but similar to that of tropical areas such as the Caribbean and East Australia (0.52 and 0.64 g C m^−2^ d^−1^, respectively; Longhurst, 1998). Our finding of low seasonal effect on sponge pumping is at odd with (laboratory) experiments conducted at high food concentrations and high sedimentation (see below). Therefore, it can be speculated that environmental factors may have smaller effect on pumping rate in oligotrophic areas with low concentrations of suspended particles (i.e., low productivity areas) such the Mediterranean and tropical seas, whereas strong effect of environmental factors cannot be disregarded in cold temperate areas with high productivity. However, it is strongly recommended that this needs to be corroborated by *in situ* measurements.

### Environmental Factors Affecting Pumping Rate

Temperature, light intensity, water flow, water viscosity, food concentration, and suspended sediment concentrations were all suggested as external factors that affect sponge pumping (e.g., Reiswig, 1971; Vogel, 1974; Frost, 1980; Riisgård et al., 1993; Riisgård and Larsen, 1995; Tompkins-MacDonald and Leys, 2008). Both ambient and artificially intensified flows have been shown to affect sponge pumping rate (Leys et al., 2011; Ludeman et al., 2017 reviewed Vogel, 1977). For some sponges, episodes of high ambient current (>15 cm s^−1^) increased the flow through the oscula by up to 7 folds (Leys et al., 2011), but theoretical considerations suggested that such passive induced flow is expected only for very slender and thin-walled sponges (mostly glass sponges) under very strong currents (Leys et al., 2011). For no slender demosponges, an increase in ambient flow resulted in constriction of the osculum and overall decrease in sponge pumping rate (Ludeman et al., 2017). At our study site, the mean water flow around the sponges was low and relatively stable, with flow >15 cm s^−1^ only occurring on occasional storms (Mendola et al., 2008) when no pumping data were collected. The mean (±SD) ambient current measured near the sponge community at 1 min intervals during five consecutive days in July 2013 was only 1.5 ± 1.4 cm s^−1^.

A high rate of sedimentation (>7 mg L^−1^) was documented to induce a reduction in sponge pumping, sometimes to a complete cessation (Reiswig, 1971; Gerrodette and Flechsig, 1979; Leys and Lauzon, 1998; Tompkins-MacDonald and Leys, 2008; Grant et al., 2018, 2019). Other than in the course of winter storms, during which no sampling took place, our oligotrophic study area was typified with clear water (visibility >10 m) and low concentrations of suspended sediment (Ribes et al., 1999b).

As the effects of strong currents and sedimentation were less relevant to our study area under common ambient conditions, we focused our analysis on the relationships between sponge pumping rate under full daylight with food abundance, temperature, and detrital particulate organic matter (POM). The seasonality we observed in the environmental factors followed the same seasonal pattern previously described for the study site by Ribes et al. (1999b). Surprisingly, as summarized in Table 4, the environmental factors had a small or negligible effect on sponge pumping.

Temperature was considered a key factor controlling physiological processes of poikilotherms, and the variation in metabolic rates over 10°C temperature increase has been used as a reference (Q_10_). Laboratory experiments and theoretical considerations indicated that sponge pumping rate should be positively correlated with temperature, which is usually reflected by a high Q_10_ (Riisgård et al., 1993). However, in our study, the estimated Q_10_ values over the natural temperature range (12–23°C) for the study species were small (ranging from 1 to 1.98), which were below the general values indicated for biological processes (between 2 and 3) and below values observed in previous studies on sponge pumping (ranging from 2.9 to 25; Reiswig, 1971; Riisgård et al., 1993). In other filter feeders, the positive correlation between temperature and pumping rate has been attributed to temperature-dependent changes in viscosity (Jørgensen et al., 1990; Riisgård and Ivarsson, 1990; Riisgård and Larsen, 2007; Larsen and Riisgård, 2009). Surprisingly, observed pumping rates showed very little correlation to seawater viscosity (Table 4, Figure 3).

Food availability has been demonstrated to affect the pumping rate of sponges and other suspension filter feeders such as polychaetes, bivalves, ascidians, and bryozoans (Winter, 1973; Frost, 1980; Navarro and Winter, 1982; Robbins, 1983; Stuart and Klumpp, 1984; Riisgård and Ivarsson, 1990; Riisgård, 1991; Petersen and Riisgård, 1992; Petersen et al., 1999; Schuster et al., 2019). These studies observed a pattern characterized by a decrease in pumping rate with the increase in food concentration that was attributed to a regulation of food uptake, in such a way that the number of filtered particles remained constant as a protection against overloading of the feeding system (Winter, 1973; Navarro and Winter, 1982; Robbins, 1983; Riisgård and Ivarsson, 1990; Riisgård, 1991; Petersen and Riisgård, 1992; Petersen et al., 1999). A similar effect of food concentration on feeding was observed in two laboratory studies conducted on sponges in which the specimens were exposed to high bacteria or yeast concentrations (from 10^6^ up to 10^9^ bacteria cell ml^−1^; Frost, 1980; Stuart and Klumpp, 1984).

Under the natural range of pico-and nanoplankton concentrations observed in the field, there was no evidence for a reduction in pumping with relation to food concentration (Table 4), and no such trend was observed in a former detailed study (Ribes et al., 1999a). It should be noted that food concentrations used in reported laboratory experiments were at least 10 times higher than the natural food concentration observed throughout the two annual cycles examined in this study (pico- and nanoplankton abundance ranged from 1.4 to 4.6 × 10^5^ cell ml^−1^). Therefore, a significantly negative effect of microbial cells concentration on pumping seems unlikely under the current natural concentration of pico-and nanoplankton cells in such oligotrophic habitat (Ribes et al., 1999b, this study).

Concentrations of suspended detrital POM were also relatively low, commonly in the range of 0.1 mg L^−1^ and rarely exceeded 0.3 mg L^−1^. Nevertheless, the abundance of detrital organic particles (>0.7 μm) was observed to negatively affect the pumping rate of all five sponge species although this reduction was significant only for *P. ficiformis* and *A. oroides*. Such reduction can potentially serve as a response to avoid clogging of the canal system and points to a higher sensitivity of these two species to detrital POM.

Taken together, our results suggest that environmental factors may be less prominent than intrinsic biological factors such as reproduction state, microbial composition, and size in controlling the pumping rates of the studied sponges.

Gametogenesis and spawning can potentially affect the rate of pumping as sponge gametes tend to develop within the sponge pumping units (the choanocyte chambers), converting them into reproductive cells (gametocytes; Bergquist, 1978; Simpson, 1984). Different studies reported a disruption of regular sponge histology, called mesohyl disruption, which consists in the disorganization of the canal system due to the occupation of choanocyte chambers by reproductive cells during the gametogenesis (Tanaka-Ichihara and Watanabe, 1990; Tsurumi and Reiswig, 1997; Ereskovsky and Gonobobleva, 2000; Riesgo and Maldonado, 2008). Sexual elements in some sponges can occupy up to 20% of the parent volume (Reiswig, 1973; Elvin, 1979; Di Camillo et al., 2012). In *C. reniformis*, for instance, this phenomenon has been documented to cause a reduction in the number of choanocyte chambers (Riesgo and Maldonado, 2008) and as pumping rate is positively correlated with the number of choanocytes (Massaro et al., 2012), a reduction in their density during the gametogenesis could lead to a decrease in the sponge pumping rate. This hypothesis is supported by evidence of a reduction in pumping activity during the reproductive cycle in the temperate sponge *C. crambe* and the tropical sponge *Svenzea zeai* species that was attributed by Turon and co-workers to a disorganization of the aquiferous system after larvae release (Turon et al., 1999; López-legentil and Turon, 2010).

The reproductive investment and spawning in most Mediterranean sponges occur between spring and fall (Coma et al., 2000; Mariani et al., 2005). *C. reniformis*, *D. avara*, and *C. crambe* reproduce from June to August (Mariani et al., 2005; Riesgo and Maldonado, 2008; de Caralt et al., 2018), *A. oroides* reproduces from June to July (Liaci and Sciscioli, 1975), and the onset of oogenesis in *P. ficiformis* has been observed in May although spawning occurs in late autumn-early winter (November–December, Maldonado and Riesgo, 2009). Unfortunately, we did not collect histological samples in this study.

The overlap of the period of higher temperature (and reduced viscosity) with that of reproduction suggests that these factors may counterbalance each other and jointly act to stabilize the pumping rate in the field. To assess the role of the reproductive cycle in the regulation of pumping rate in sponges, the density of functional choanocytes and choanocyte chambers should be examined throughout the year.

### Species Comparison

Averaging sponge pumping rate values normalized to sponge size (i.e., volume or mass) was commonly used to characterize sponge specific pumping rate and to compare it among sponge species (e.g., Riisgård et al., 1993; Ribes et al., 1999a; Weisz et al., 2008; McMurray et al., 2018). The mean pumping rate values observed in this study were within the range of those reported in the literature for temperate and tropical species. The annual average estimated in this study varied from 2 ml min^−1^ (cm sponge)^−3^ for *Petrosia ficiformis* to 16 ml min^−1^ (cm sponge)^−3^ by *Crambe crambe*. These values are within the reported range of dye-based pumping rate estimates from 1.2 ml min^−1^ (cm sponge)^−3^ by *Spongia* sp. (Tsubaki and Kato, 2014) to 35 ml min^−1^ (cm sponge)^−3^ by *Callyspongia plicifera* (Weisz et al., 2008).

However, the use of such mean values may be misleading as this method may lead to very different results, pending on the size of the specimens studied. Indeed, an increase in sponge size has long been observed to negatively affect specific pumping rate (e.g., Reiswig, 1974; Frost, 1980). The large variation among species in the rate of reduction of size-specific pumping rate with size (with exponents ranging from −0.2 to −0.7 in this study) resulted with large differences (3–33 folds, Table 5, Figure 5) in the size-specific pumping rates. Such variation implies that the comparison among species becomes largely affected by the size of the examined specimens as illustrated in Figure 5.

For example, small specimens (sponge volume 3 cm^3^) of *C. crambe*, *D. avara*, and *C. reniformis* pumped at a similar rate (volume normalized, Figure 5). By contrast, the specific pumping rates of medium size specimens (sponge volume 32 cm^3^, Figure 5) were markedly different. Similarly, at a small size (sponge volume 3 cm^3^; Figure 5), the size-specific pumping rate of *A. oroides* was higher than that of *P. ficiformis*. By contrast, at large size (sponge volume 316 cm^3^; Figure 5), the size-specific pumping rate of *P. ficiformis* was higher than that of *A. oroides*. Therefore, we conclude that due to the strong and species-dependent effect of size on volume-specific pumping rate, the use of single size normalized value to characterize and compare sponge species is questionable.

Our study points to the importance of considering specimen size for comparative metabolic and ecological studies. For instance, in this study, *D. avara* exhibited an order of magnitude higher pumping rate than that reported 20 years earlier by Ribes et al. (1999a) [0.44 vs. 0.02 L s^−1^ (L sponge)^−1^, respectively]. This apparent discrepancy could be mistakenly attributed to methodological or decadal differences, but in practice, it is simply reconciled by considering the allometry and the size of the sampled specimens (0.01–0.6 g AFDW in this study vs. 0.3–2.3 g AFDW in Ribes et al., 1999a). Similarly, the mass-specific pumping rate of *Halichondria panicea* was estimated to be five times higher in the explants examined by Kumala et al. (2017) than previous studies (Riisgård et al., 1993). This difference is likely the result of the smaller size specimens (0.018 cm^3^) used by Kumala and colleagues in 2017 compared to the size range used by Riisgård and colleagues in 1993 (4–17 cm^3^).

The size of the studied sponges must be taken into consideration when comparing sponge metabolic rates, because differences in the size of the studied animals may lead to very different conclusions.

### High and Low Microbial-Abundance Sponge Pumping Rate

HMA sponge species have been described to have a lower volume-specific pumping rate than LMA sponge species (Weisz et al., 2008). However, analysis of Weisz and collaborators original data indicated that the average size of the HMA sponges that they sampled was 15 ± 14 L, ~50 folds larger than the average size of the LMA species 0.29 ± 0.25 L (data courtesy of Jeremy Weisz).

The species we studied were classified as HMA (*Chondrosia reniformis*, *Petrosia ficiformis*, and *Agelas oroides*) and LMA species (*Dysidea avara* and *Crambe crambe*; Vacelet and Donadey, 1977; Bjork et al., 2013; Gloeckner et al., 2014 and references therein) based on electron microscopy. Although we examined only a few species of each category, our results may provide some insight into the validity of the distinction between HMA and LMA sponge species. In the seasonal study, the volume-specific pumping rate of the three HMAs examined ranged from 2.0 to 3.5 ml min^−1^ (cm sponge)^−3^ and 8.5 to 16.5 ml min^−1^ (cm sponge)^−3^ for the two LMAs. In accordance with Weisz et al. (2008), the average pumping rates (Supplementary Table S2) indicated that HMA species pumped ~80% less water per unit of volume than LMA species. Inadvertently, as in Weisz study, the HMA “small, single-osculated sponge specimens” we used for the seasonal study were on average 2–13 times larger than the LMA specimens (Table 1) and interspecies size difference alone accounted for the entire difference in volume-specific pumping rates observed between HMA and LMA sponges.

Clearly, the allometric constants of more HMA and LMA species should be studied before any generalization is made. However, at this stage, it seems plausible to assume that differences in pumping rate between both strategies could be related to a larger effect of size in reducing specific pumping rate in HMA than in LMA species. This would be consistent with the longer and narrower water canals in HMA sponges than LMAs (Vacelet and Donadey, 1977).

## Conclusions

The strong seasonality of our study area provided an excellent opportunity to examine the effects of the seasonal variation of environmental factors on the metabolic activity of benthic organisms. Surprisingly, the natural variation of food resources, temperature, and physiological conditions had a small effect on the pumping rate of five dominant Mediterranean sponge species examined *in situ* over two annual cycles. Further studies are required to determine whether a lack of strong seasonal effect on sponge pumping rates occurs in other regions marked by strong seasonality and what are the mechanisms underlying this phenomenon.

Our results together with those from the literature indicated that sponge-specific pumping rate tends to decrease with size and that the magnitude of this effect differs markedly among species. In this regard, the common use of a “standard sponge” as a tool to compare the pumping rate among sponge species and to conduct sponge population and community quantifications (e.g., Yahel et al., 2007; Leys et al., 2011) is questionable and may lead to erroneous results. Therefore, in order to characterize sponge species pumping rate and to properly quantify the volume of water processed by sponge populations, we propose that the assessment of pumping rates should be carried over the size distribution of the population of each species.

Since sponge respiration, feeding, excretion, and reproduction are all mediated by the water they filter, a better comprehension of the factors that control sponge pumping rate is essential for understanding the basic physiology of marine sponges and the processes that they are mediating in benthic communities.

## Data Availability Statement

The raw data supporting the conclusions of this manuscript will be made available by the authors, without undue reservation, to any qualified researcher.

## Author Contributions

TM, MR, GY, and RC designed the study, performed the experiments, and analyzed the data. All authors contributed critically to the draft and gave the final approval for publication.

### Conflict of Interest

The authors declare that the research was conducted in the absence of any commercial or financial relationships that could be construed as a potential conflict of interest.
