# Supplementary material for: Size Is the Major Determinant of Pumping Rates in Marine Sponges
Source: Front Physiol. 2019 Dec 11;10:1474. doi: 10.3389/fphys.2019.01474 (PMC6917621; doi:10.3389/fphys.2019.01474)
Supplement: Supplementary file 1 [file Data_Sheet_1.PDF]

Supplementary material

Figure S1. Variation in SE as a proportion of the mean with sample size (No. of the specimen) to determine the minimum sample size for mass-specific pumping rate in a particular session.

Figure S2. Pumping rate (PR, mL min^{-1}) vs. Biomass (AFDW, g) on multi-osculated specimens.

Figure S1

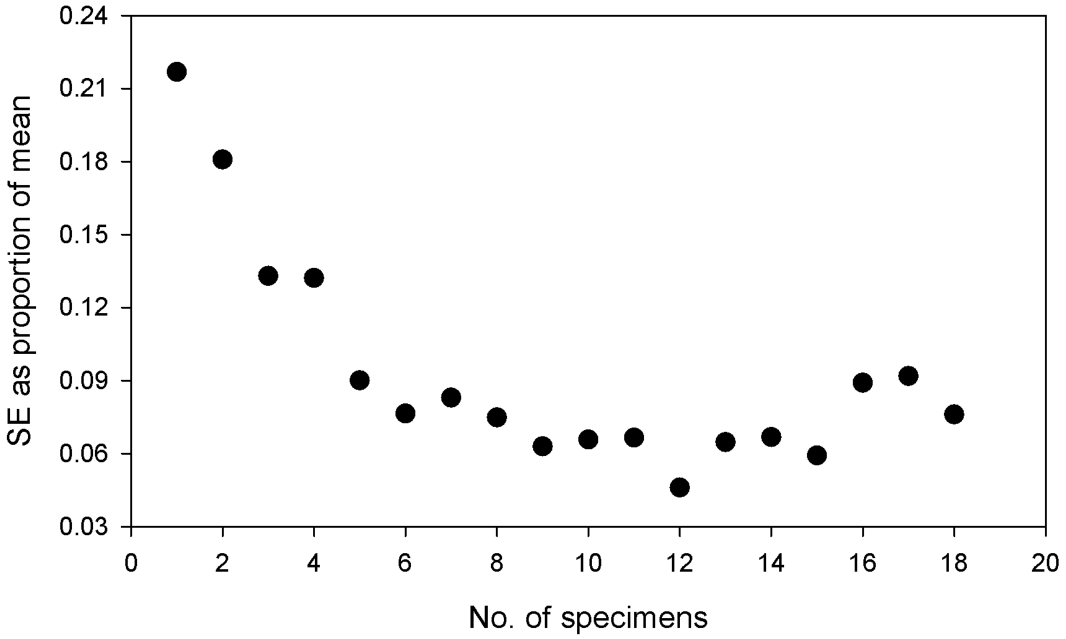
0.24
0.21
0.18
0.15
0.12
0.09
0.06
0.03
SE as proportion of mean
0
2
4
6
8
10
12
14
16
18
20
No. of specimens

Figure S2

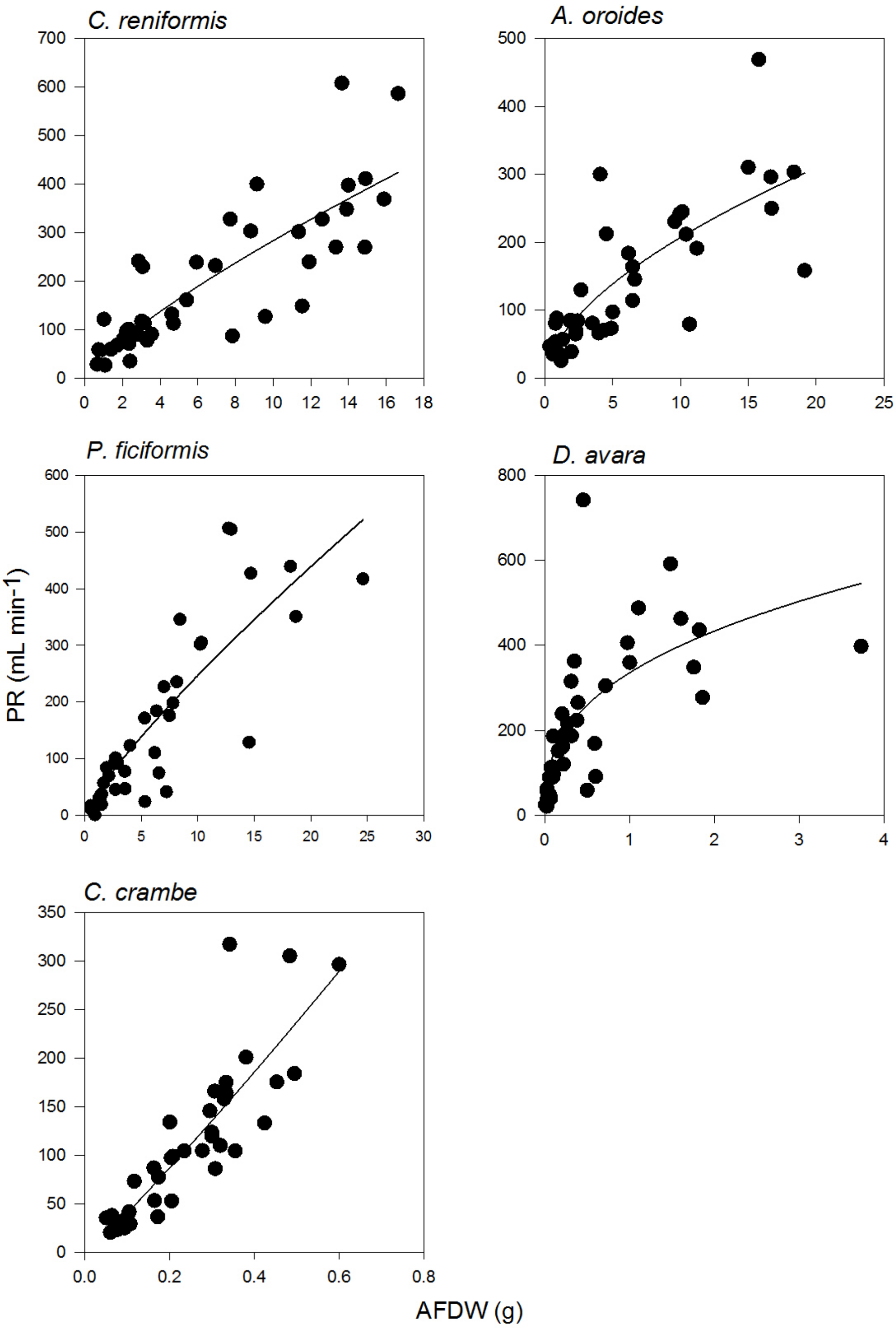
C. reniformis
A. oroides
P. ficiformis
D. avara
C. crambe
PR (mL min-1)
AFDW (g)

Table S1. Comparison between volume-specific pumping rate (PR_v, mL min^{-1} (cm sponge)$^{-3}$) measured using the dye front speed (DFS) and dye speed (DS) method for the same specimens at the same time. Data are expressed as mean ± 95% CI. *n* number of sampled specimens; *p* values are for a paired t-test. The statistical power to detect a 20% difference from the DFS method was calculated for each species using the observed variability of the paired differences and α=0.05.

Specie	n	DFS	DS	*p*-value	Statistical power	Deviation of DS from DFS
C. reniformis	19	4.46 ± 0.97	4.36 ± 0.97	0.828	100%	-3%
P. ficiformis	20	2.41 ± 0.97	2.14 ± 1.20	0.488	79%	-11%
D. avara	21	14.42 ± 4.32	13.60 ± 3.48	0.813	90%	-6%
A. oroides	22	2.48 ± 0.97	2.01 ± 0.82	0.413	96%	-19%
C. crambe	17	25.13 ± 10.56	17.08 ± 7.60	0.107	79%	-32%

Table S2. Average volume-specific pumping rate (PRv, mL min^{-1} (cm sponge)$^{-3}$) normalized by sponge volume measured *in situ* during each sampling session. Data expressed as mean ± 95% CI. *n*, number of sampled specimens for each sampling session.

Sampling date	Temp (°C)	*A. oroides*			*C. reniformis*			*P. ficiformis*			*D. avara*			*C. crambe*		
		n	PRv	95%CI	n	PRv	95%CI	n	PRv	95%CI	n	PRv	95%CI	n	PRv	95%CI
Jul-12	22	7	2.2	0.6	14	2.8	0.5	9	2.7	1.3	7	3.3	2.2	8	9.2	2.2
Oct-12	19	16	2.3	0.8	56	4.2	0.5	7	1.2	0.5	15	5.8	1.5	18	13.1	2.8
Nov-12	16	15	2.1	0.6	17	3.3	0.5	15	2.1	0.5	11	9.8	5.1	15	15.8	3.0
Jan-13	13	13	1.9	0.5	24	4.0	1.2	15	2.0	0.5	11	7.4	4.1	22	18.5	3.7
Apr-13	13	16	1.7	0.8	16	2.9	0.9	11	2.1	1.2	14	8.0	2.9	16	12.4	3.3
May-13	15	12	1.5	0.9	17	2.1	0.4	14	1.5	0.6	13	9.1	4.0	16	19.6	4.7
Jul-13	21	19	1.3	0.4	15	3.0	1.1	18	1.5	0.3	15	9.0	2.9	21	15.0	3.3
Oct-13	19	16	2.7	1.0	15	4.6	1.6	15	1.2	0.5	14	10.9	3.8	17	15.0	5.5
Jan-14	13	14	2.6	1.0	15	3.7	1.3	15	1.6	0.5	8	9.6	3.9	13	26.0	13.4
Apr-14	15	16	1.5	0.4	17	4.0	1.1	13	2.6	1.5	16	10.1	4.5	18	15.5	5.8
Jul-14	22	14	3.0	0.9	15	4.1	0.9	15	2.9	0.7	20	10.0	2.6	17	21.7	5.7
**Average**	**17.1**	**14**	**2.1**	**0.7**	**20**	**3.5**	**0.9**	**13**	**2.0**	**0.7**	**13**	**8.5**	**3.4**	**16**	**16.5**	**4.8**
**95%CI**	**2**	**-**	**0.3**	**-**	**-**	**0.4**	**-**	**-**	**0.3**	**-**	**-**	**1.3**	**-**	**-**	**2.7**	**-**
